# Systematic and Individualized Preparation of External Ear Canal Implants: Development and Validation of an Efficient and Accurate Automated Segmentation System

**DOI:** 10.3390/jimaging11080264

**Published:** 2025-08-08

**Authors:** Yanjing Luo, Mohammadtaha Kouchakinezhad, Felix Repp, Verena Scheper, Thomas Lenarz, Farnaz Matin-Mann

**Affiliations:** 1 Department of Otorhinolaryngology, Head and Neck Surgery, Hannover Medical School, 30625 Hannover, Germany; yanjing.luo@mh-hannover.de (Y.L.); kouchakinezhad.mohammadtaha@mh-hannover.de (M.K.); scheper.verena@mh-hannover.de (V.S.); lenarz.thomas@mh-hannover.de (T.L.); 2 Lower Saxony Center for Biomedical Engineering, Implant Research and Development (NIFE), Hannover Medical School, 30625 Hannover, Germany; 3 Cluster of Excellence “Hearing4all”, German Research Foundation, Hannover Medical School, 30625 Hannover, Germany; 4 OtoJig GmbH, Feodor-Lynen-Straße 35, 30625 Hannover, Germany; repp.felix@otojig.com

**Keywords:** external ear canal implant (EECI), image segmentation, customized implants, shape, computed tomography, 3D slicer, 3D printing

## Abstract

External ear canal (EEC) stenosis, often associated with cholesteatoma, carries a high risk of postoperative restenosis despite surgical intervention. While individualized implants offer promise in preventing restenosis, the high morphological variability of EECs and the lack of standardized definitions hinder systematic implant design. This study aimed to characterize individual EEC morphology and to develop a validated automated segmentation system for efficient implant preparation. Reference datasets were first generated by manual segmentation using 3D Slicer^TM^ software version 5.2.2. Based on these, we developed a customized plugin capable of automatically identifying the maximal implantable region within the EEC and measuring its key dimensions. The accuracy of the plugin was assessed by comparing it with manual segmentation results in terms of shape, volume, length, and width. Validation was further performed using three temporal bone implantation experiments with 3D-Bioplotter©-fabricated EEC implants. The automated system demonstrated strong consistency with manual methods and significantly improved segmentation efficiency. The plugin-generated models enabled successful implant fabrication and placement in all validation tests. These results confirm the system’s clinical feasibility and support its use for individualized and systematic EEC implant design. The developed tool holds potential to improve surgical planning and reduce postoperative restenosis in EEC stenosis treatment.

## 1. Introduction

External ear canal (EEC) stenosis is a challenging disease in otology that can be caused by congenital or acquired factors, such as chronic inflammation, trauma, tumors, and surgical complications [1,2]. The incidence of EEC cholesteatoma in patients with congenital EEC stenosis is reported to be as high as 43% [3]. While medical management may be considered in some cases, surgical treatment remains the primary approach. Common surgical procedures include complete removal of the stenotic plug, canaloplasty with or without meatoplasty, re-epithelialization using split-thickness skin grafts (STSGs), and ear canal stenting [2]. However, the postoperative restenosis rate caused by persistent infection or scar tissue proliferation is frequent, with a recurrence rate up to 50% [4].

To address these issues, external ear canal implants (EECIs) have been proposed as a promising strategy. These implants provide mechanical support to prevent soft tissue overgrowth-related restenosis and, when loaded with therapeutic agents, can deliver antimicrobial drugs locally to reduce infection risk [5]. However, EEC morphology varies widely between patients and even between ears in the same patient, complicating the design of standardized EECIs [6]. This variability requires the development of individualized treatment methods, particularly in the design of customized EECIs.

Although the individuality of EEC anatomy is clinically recognized, it has rarely been quantified or systematically addressed in implant design [7]. High-resolution computed tomography (HRCT) or cone-beam computed tomography (CBCT) is often used to visualize bony structures [8], but current clinical methods for assessing the EEC rely heavily on manual segmentation. These methods are time-consuming, subject to operator variability, and inherently limited in scalability and reproducibility [9,10].

To overcome these challenges, this study introduces a custom-developed plugin for 3D Slicer^TM^ that enables automated segmentation of the EEC based on CBCT data. By incorporating both bony and partially cartilaginous portions of the canal, our method captures a more complete anatomical model. The system integrates image processing, semi-automated control-point placement, threshold-based segmentation, and implant modeling within a streamlined workflow.

The primary goal of our study was to develop and validate an efficient and accurate segmentation pipeline that enables reliable extraction of implantable regions within the EEC, suitable for individualized 3D-printed EECI fabrication. This approach not only improves segmentation speed and repeatability but also facilitates the clinical translation of personalized implants. To demonstrate its feasibility, we conducted quantitative segmentation evaluations on 25 patients (50 EECs) and performed implant fitting assessments in human cadaveric temporal bones. Our study contributes to the field by not only providing a robust framework for automated segmentation and implant fabrication but also by demonstrating the practical feasibility of these EECIs by performing comprehensive fitting tests in temporal bone specimens.

## 2. Materials and Methods

### 2.1. Study Design

This study was designed as an experimental and validation research project to develop and evaluate an automated segmentation system, i.e., HRCT scans, for the EEC. The experimental phase focused on the development of an automated segmentation plugin for the 3D Slicer^TM^, while the validation phase involved a systematic comparison between the manual and automated segmentation results, followed by the fabrication and fitting tests of 3D-printed EECIs on N = 3 human cadaver temporal bones based on these results [11].

Firstly, manual segmentation was performed using 3D Slicer^TM^ to measure the EEC and define key anatomical parameters such as length, width, and volume of the EEC and the tympanic membrane area (TM Area). To address these limitations and simplify the design process of individualized EECIs, we developed a custom plugin for 3D Slicer^TM^ to automate the segmentation process. The plugin was designed to accurately and efficiently map the complex anatomy of the EEC.

The EECs were segmented using consistent anatomical boundaries, with the zygomatic arch and mastoid defining the outer border, while the inner border followed the tympanic membrane (TM). Both manual and plugin-assisted automated segmentation methods were employed to delineate the largest implantable region of the EEC. A systematic comparison was conducted between the results of these two approaches. Furthermore, to validate the clinical feasibility of our method, the segmentation results from both methods were used to fabricate 3D-printed EECIs, which were subsequently evaluated through fitting experiments using human cadaver temporal bone specimens.

The study protocol was reviewed and approved by the Ethics Committee of Hannover Medical School (Medizinische Hochschule Hannover—MHH) (Project identification code 3699-2017). Although no direct clinical interventions were performed on patients, this study used anonymized cone-beam computed tomography (CBCT) imaging data from previous clinical cases. Furthermore, all experiments involving human cadaver temporal bones were performed in accordance with institutional guidelines and registered under the number 9236_BO_K_2020. This study was conducted in accordance with the principles of the Declaration of Helsinki, and informed consent was waived due to the retrospective and non-interventional nature of this study.

### 2.2. Patient Selection and Data Acquisition

This study analyzed the EEC using CBCT imaging data. In total, 25 patients were selected based on strict inclusion and exclusion criteria. The inclusion criteria required that both EECs be clearly visible in the Digital Imaging and Communications in Medicine (DICOM) datasets obtained from the CBCT scans. Patients with a history of EEC disease, previous surgeries, or congenital malformations affecting the EEC were excluded from this study to eliminate any confounding factors that could affect segmentation accuracy and subsequent analyses.

CBCT was selected for its high spatial resolution and suitability for bone imaging. All patients were scanned by a clinical 3D ACCUITOMO 170 Digital CBCT scanner (J. Morita Tokyo mfg. Corp., Tokyo, Japan), with a tube voltage of 89.0 kV, a tube current of 6.9 mA, and an exposure time of 17.5 s. The isotropic voxel size was set to 0.25 mm, ensuring adequate spatial resolution for segmentation. The dose area product (DAP) was 2540 mGy·cm^2^, following standard clinical guidelines to balance image quality and radiation exposure. Scans were performed using a standardized protocol with slice thicknesses and voxel sizes optimized for high-resolution imaging of bone structures (slice thickness: 0.5 mm; voxel size: 0.2 × 0.2 × 0.2 mm^3^). These parameters ensured consistent data quality, which is critical for both manual and automated segmentation processes. The DICOM datasets were anonymized before analysis to protect patient privacy.

### 2.3. Manual Segmentation Process

**Figure 1:**
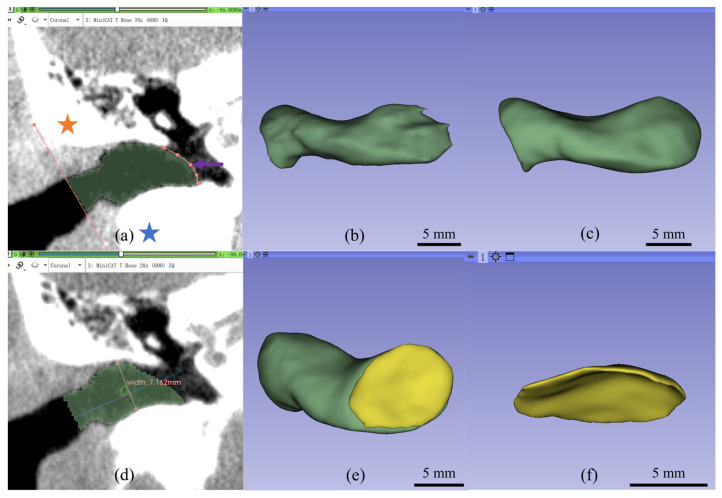
Overview of the manual segmentation process for the external ear canal (EEC) using 3D Slicer. (**a**) Coronal plane view with a straight line connecting the zygomatic arch and mastoid defining the external boundary, a curve outlining the tympanic membrane as the internal boundary, and the segmented EEC region between the boundaries. The orange star indicates the location of the zygomatic arch, the blue star indicates the location of the mastoid’s defining point, and the curve pointed by the purple arrow indicates the location of the tympanic membrane. (**b**) A 3D model of the EEC generated after segmentation in the coronal plane. (**c**) Smoothed 3D model of the EEC after boundary refinement in the axial plane. (**d**) Coronal plane view of the “short axis,” defined as the line segment perpendicular to the long axis, passing through its midpoint and representing the width of the EEC. (**e**,**f**) Segmentation and visualization of the tympanic membrane model.

Manual segmentation of the EEC was performed by a researcher with clinical experience in otolaryngology using 3D Slicer^TM^ version 5.2.2. (http://www.slicer.org, accessed on 22 February 2023) (Surgical Planning Laboratory, Brigham and Women’s Hospital, Harvard Medical School, Boston, MA, USA) (Figure 1).

Initially, the DICOM images were adjusted in the coronal plane to ensure clear visualization of the right EEC and TM. The line connecting the zygomatic arch and mastoid process was determined as the external boundary, and the TM was designated as the internal boundary (Figure 1a). The EEC was segmented layer by layer in the coronal plane, and a 3D model of the EEC was generated upon completion (Figure 1b). Subsequent refinement was performed in the axial plane to further delineate each segmented layer and smooth the surface of the 3D model (Figure 1c).

Second, the length and the width of the EEC were measured. The longest segment on the coronal plane was designated as the external boundary surface. The junction between the handle of the malleus and the TM, known as the umbo, was determined as the midpoint of the internal boundary surface. The length of the EEC was defined as the distance between the midpoint of the external and internal boundaries. This line was then used to draw a vertical line at the midpoint of this line in the coronal plane, which was defined as the width of the EEC (Figure 1d).

The volume of the EEC was calculated using the model function in 3D Slicer^TM^. Furthermore, to measure the surface area of the TM, a single layer at the TM surface was re-segmented to ensure that the thickness was only one layer (Figure 1e,f). The model function was then used to determine the surface area of this layer, which was halved to estimate the surface area of the TM.

### 2.4. Development of the Segmentation and Implant Creation Plugin

**Figure 2:**
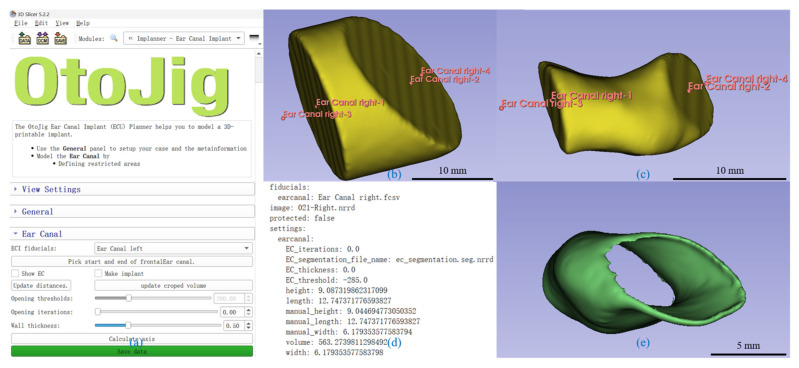
Workflow of the developed plugin for automated EECI generation. (**a**) The graphical user interface of the custom plugin, displaying key input parameters and controls. (**b**) Visualization of the four control points used to define the initial cylindrical model for the external auditory canal (EEC). (**c**) Segmentation of the EEC model based on the adjustable threshold slider, enabling precise boundary determination. (**d**) Automated calculation and display of quantitative measurements, including volume, height, width, and length, generated by the plugin. (**e**) The final 3D-printed implant model created by the plugin, ready for evaluation and surgical application.

A plugin for 3D Slicer^TM^ for automated segmentation was developed to accurately and efficiently model the shape of the EEC for individualized implant design (Figure 2).

The EEC’s shape was approximated as a cylinder. The cylinder walls, defined by bony structures, came along with significant intensity changes between air and bone and were readily identified using thresholding techniques. However, the base surfaces presented greater challenges for segmentation. On the medial side, the TM was often identified in CBCT scans but was difficult to segment automatically. On the lateral side, there was no image contrast to delineate structures.

Anatomical Initialization and Volume of Interest Restriction

To address these challenges, we segmented the EEC by cutting the cylindrical shape with two tilted planes defined by four control points. Points A and B represented the locations of the planes and were manually placed in the coronal plane. Point A was positioned at the outer edge (proximal to the EEC), and Point B was positioned at the inner edge (near the TM). Points C and D defined the normal of the planes and determined the lateral extent of the initial cylindrical shape. Point C was placed lateral to Point A in the coronal plane, while Point D was placed medial to Point B. These points and an initial diameter of 20 mm defined a truncated cylindrical volume of interest (VOI), which served as the starting model for segmentation (Figure 2b). Limiting the VOI speeds up the following steps and helps with an interactive visualization.

Threshold Selection and Air–Bone Interface

The plugin provided a threshold slider to segment the air (or optionally the soft tissue) within the EEC. A Gaussian smoothing filter, with a sigma equal to the mean voxel size (in our case, 0.3 mm) and otherwise default parameters, reduced noise in the image data and smoothed transitions between high- (bone) and low-intensity (air) regions. Since low-intensity voxels were not only present within the EEC, a connected component analysis was performed using scipy’s label method with a 3D 6-neighborhood on the threshold VOI. For each component, the mean distance of all voxels was calculated, and only the component closest to the line connecting Points A and B was used. Therefore, it was important that the defined planes cut the peripheral surface given by the bony structures; otherwise, non-EEC regions (e.g., parts of the middle ear) might erroneously have been included.

Handling Limited Soft Tissue Contrast

CBCT contrast does not reliably distinguish soft tissue. The plugin implicitly approximates the medial base plane near the TM through the tilted control-plane setup, focusing on segmenting the soft-tissue/bone interface. Since the Gaussian filter created a smooth transition between low- and high-intensity values, changing the threshold within a certain range was used to fine-tune the exact position of the lateral surface of the EEC’s segmentation, optimizing the implant’s size based on the surgeons’ preferences for a looser or tighter fit. This hybrid method accommodates anatomical consistency despite imaging limitations.

Three-Dimensional Model Verification and Surface Metrics

The generated 3D model was finally verified for accuracy based on the coronal and axial planes (Figure 2c). The plugin calculates the volume, length, width, and height. The volume was calculated using the number of voxels and dimensions. For the dimensions, the plugin calculated the center of both boundary planes and used the distance between these centers as the length. The plugin further changed the view such that the sagittal plane was orthogonal to this length and in the middle of it. In this view, the longest line in the cross-section of the segment on this plane passing through the midpoint was defined as the width, and the perpendicular height of the segmented cross-section was measured.

Implant creation and Export

For implant modeling, the EEC segmentation was hollowed (Figure 2c) using a distance transformation to the surface to define the wall thickness. The base sides were then trimmed, leaving only the cylindrical walls, thus reducing the length of the implant by two times the wall thickness compared to the segmentation (i.e., typically 1 mm). All the described steps were performed on voxel data. Finally, the plugin automatically sets the binary image, representing the implant as a label map in the segmentation module of 3D Slicer, which allows graphical representation and exports it as an STL file to support 3D printing.

Implementation and Inter-operator Variability

The segmentation algorithm was implemented in Python version 3.9.10 and integrated into the 3D Slicer^TM^ platform using the extension wizard. Image-based algorithms were implemented using the ndimage tools of scipy [12]. The 3D Slicer’s graphical user interface was used to manipulate the location of the control points, and sliders were used for parameters like the threshold and wall thickness. Changing any of these triggered a re-evaluation of the algorithm, which then updated the label map and therefore the graphical representation. Although control-point placement is manual, the plugin constrains segmentation within a truncated cylindrical VOI, and it performs automatic component selection based on proximity to anatomical landmarks. These steps help localize the segmentation and provide a consistent starting point across users. However, the plugin is designed as an interactive tool, and minor variability may occur depending on clinical preference or anatomical interpretation.

The user interface was designed to be intuitive (Figure 2a), allowing clinicians to easily place the control points, adjust the cylinder density, and visualize the segmented model in real time. The plugin also provided an automated calculation of the segmented EEC’s dimensions, thus streamlining the workflow for custom implant design (Figure 2d).

### 2.5. Validation of Segmentation Accuracy

To evaluate the accuracy and efficiency of the automated segmentation plugin, a comparative analysis was performed between the automated and manual segmentation methods.

Segmentation Process and Time:

Manual segmentation was performed to segment the EEC in all 25 patients (50 ears), which was carried out by an experienced clinician using 3D Slicer^TM^ 5.2.2. Given that the implant is a hollow tubular structure confined by the bony EEC, we used the interface between the bony wall and the TM as the internal segmentation boundary and the line connecting the zygomatic processes and mastoid processes in the coronal plane as the external boundary. By using the two defined internal and external segmentation boundaries, we performed manual segmentation to segment the maximum implantable portion. After completing the segmentation, we recorded the time required for each manual segmentation, and we measured the volume, length, and width from the manually segmented models for comparison. Subsequently, the same 50 ears were segmented automatically using the same internal and external boundaries. The time required for each segmentation (i.e., each ear) was recorded. The automated plugin generated a 3D model for each EEC and provided measurements of volume, length, and width.

Three-Dimensional Model Overlap and Shape Consistency:

For each ear, the 3D models generated by the manual and automated segmentation were spatially overlaid to assess shape consistency. Models were aligned using rigid body transformation to ensure spatial consistency. This alignment was performed using Lumafield and 3D Slicer^TM^, and the degree of overlap was quantified using the Dice Similarity Coefficient (DSC), with values closer to 1 indicating higher similarity between the models. Additionally, the 95th percentile Hausdorff Distance (HD 95%) was used to measure the spatial deviation between the two segmentation boundaries, providing a robust metric that minimizes the influence of outliers. Together, these metrics comprehensively evaluate both volumetric agreement and boundary precision between the segmentation methods.

Statistical Analysis: Statistical comparison used paired *t*-tests (Section 2.7).

### 2.6. Ex Vivo Comparative 3D-Printed EECI Implantations in Human Cadaver Temporal Bones

Three-Dimensional Model Preparation and Printing:

To evaluate the fitting accuracy of the implants designed using both segmentation methods, we used human cadaver temporal bones. Three human cadaver temporal bones were CBCT-scanned to obtain high-resolution DICOM datasets. These datasets were imported into 3D Slicer^TM^ to generate the implants.

Both manual and automated segmentation methods were used to create 3D models of the EEC implants. These models were exported in Standard Tessellation Language (STL) format for 3D printing.

The implants were 3D-printed using a 3D-Bioplotter^®^ Manufacturers Series (Desktop Health, Gladbeck, Germany), a high-precision extrusion-based bioprinter suitable for medical-grade materials. A medical grade UV-curable silicone elastomer (60A MG, BIO-83-6001, Momentive Performance Materials Inc., Waterford, NY, USA) was used due to its flexibility and biocompatibility. The material was extruded through a 0.41 μm nozzle at a pressure of 1.2 bar and a printing speed of 2 mm/s, with a layer thickness of 0.38 μm. The implants were subsequently cured under UV light at 365 nm with an intensity of 1800 × 100 μJ/cm^2^ for 30 min, ensuring proper cross-linking and mechanical stability. This approach follows previous studies utilizing UV-curable silicone in medical applications [5].

Fitting Experiments of EECI Implantations in Temporal Bone Specimens:

Two otolaryngologists performed fitting accuracy experiments of the 3D-printed implants in the temporal bones. Prior to insertion, each implant was visually inspected for potential printing defects or inconsistencies. The temporal bones and especially the EEC were carefully examined using an endoscope (Karl Storz Endoscope Tele Pack+ TP101, Tuttlingen, Germany). The EEC was cleared of cerumen, and any fluid was aspirated to ensure unobstructed access. After confirming the EEC’s patency, the implant was grasped using forceps and inserted under microscopic guidance (OPMI PROergo S7, Carl Zeiss Meditec, Jena, Germany) while timing the procedure. After implantation, the internal fit of the implant was examined with an endoscope to assess the alignment between the implant’s internal portion and the TM. Visual inspection was performed to detect any visible gaps or misalignments between the implant’s outside portion and the bony edges of the EEC.

The assessment criteria include the following: (1) handling difficulty rated on a 1–6 scale, where 1 indicates the easiest handling with forceps and 6 the most challenging; (2) implantation time (in seconds), measured from the start of insertion to proper placement and alignment; (3) fitting accuracy evaluated under endoscopic observation, rated on a 1–6 scale (1 for excellent fit and 6 for poor fit); and (4) fitting accuracy quantified by post-implantation micro-CT imaging, rated on a 1–6 scale (1 for excellent fit and 6 for poor fit).

Evaluation of Fitting Accuracy:

After implantation, the temporal bone specimens were scanned using a 3D ACCUITOMO 170 Digital CBCT scanner (J. Morita Tokyo mfg. Corp., Tokyo, Japan) to assess the internal fitting accuracy. The generated DICOM data were imported into 3D Slicer^TM^ (version 5.2.2) for segmentation and 3D model reconstruction.

Fitting accuracy was quantified by comparing the implant surface with the surrounding bone using point cloud analysis. The root mean square (RMS) distance between surfaces was calculated to assess consistency.

Statistical Analysis:

The fitting accuracy of the 3D-printed implants was assessed using model overlap analysis, followed by statistical comparisons, as detailed in G.

### 2.7. Statistical Analysis

To evaluate the performance of the automated segmentation plugin compared to manual segmentation, a comprehensive statistical analysis was performed as follows:

Data Collection and Preparation: EEC volume, length, and width and TM area were measured in 50 ears (25 binaural patients) using a manual segmentation method. For the maximum implantable portion of EEC, the volume, length, width, and segmentation time were measured using both automated and manual segmentation methods.

The data were recorded and organized into two datasets corresponding to each segmentation method.

Descriptive Statistics: Descriptive statistics, including mean, standard deviation, and range, were calculated for each measurement in both segmentation methods. This provides a summary of the central tendency and dispersion of the measurements.

Comparison of Segmentation Methods: Paired *t*-Test: Measurements were analyzed using paired *t*-tests to compare manual and automated methods. This test assesses whether there is a statistically significant difference between the volume, length, and width means of the two related groups.

Significance Threshold: The significance level was set at 0.05. A *p*-value greater than 0.05 indicated no significant difference between the methods, indicating that both methods produced similar results.

Three-Dimensional Model Fitting Accuracy: Point Cloud Registration: Use the point cloud data from the 3D scan to assess the fit accuracy of the implants. Cloud Compare was used to align and compare the 3D model of the implant with the 3D model of the temporal bone specimens.

Quantitative Metrics: The deviation between the implant model and the temporal bone specimens was measured to assess the accuracy of the fit. The metric is RMS, which is used to assess consistency. The RMS distance between surfaces was analyzed by using CloudCompare 2.13.2. Statistical analysis of RMS values was performed using paired *t*-tests in GraphPad Prism 8.

Software Used: All statistical analyses, including paired *t*-tests and descriptive statistics, were performed using GraphPad Prism 8. This software was chosen because of its comprehensive statistical functionality and ease of use in data analysis.

Interpretation of Results: Statistical evaluation of results: The results of the paired *t*-tests were used to determine whether there was a significant difference between the automated and manual methods. A *p*-value of less than 0.05 indicates a significant difference, while a *p*-value greater than 0.05 indicates no significant difference, which means that the two methods are comparable

## 3. Results

### 3.1. Variability in the EEC Measurements

In this study, the CBCT imaging of temporal bones from 25 patients (11 male and 14 female) with a mean age of 58 years (min 19 years old; max 92 years old) was analyzed, including both left and right EECs (N = 50). Key anatomical parameters of the EEC, including length, width, volume, and TM area, were measured. The Gaussian distribution was confirmed. Statistical analysis was conducted using analysis of variance (ANOVA) to assess variability across different subgroupings. First, data were stratified by side, where all measurements were compared separately for left and right EECs. Second, data were grouped by sex, comparing segmentation measurements within male and female patient subgroups. Statistical analysis accounted for these pairings to ensure valid comparisons.

The analysis results showed that there were significant morphological differences within individuals (side group) and between individuals based on gender (sex group). The EEC length ranged from 10.75 mm to 19.99 mm (mean: 15.91 mm), while the width varied between 3.634 mm and 11.06 mm (mean: 7.262 mm). The mean volume is 568.43 mm^3^ (min 308.82 mm^3^; max 999.7 mm^3^). And the TM area ranged from 49.56 mm^2^ to 88.275 mm^2^ (mean: 67.755 mm^2^).

When grouped by side, the mean EEC length was 16.22 ± 2.23 mm in the right ear and 15.61 ± 1.79 mm in the left ear, the mean EEC width was 7.273 ± 1.561 mm in the right ear and 7.252 ± 1.514 mm in the left ear, and the mean EEC volume was 574.81 ± 107.12 mm^3^ in the right ear and 562.04 ± 126.43 mm^3^ in the left ear, while the mean TM area was 68.36 ± 7.91 mm^2^ in right ear and 67.15 ± 8.85 mm^2^ in left ear.

**Figure 3:**
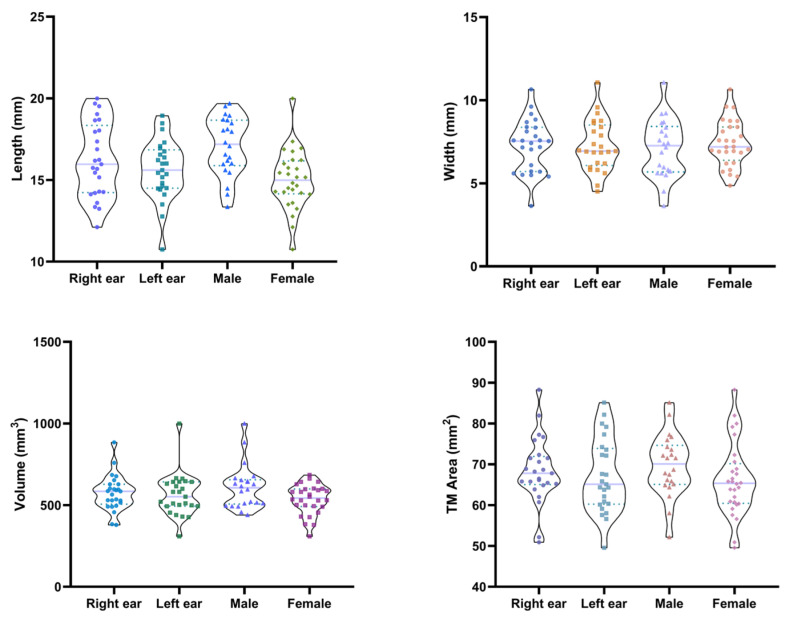
Distribution of the EEC length, width, and volume and TM area.

When grouped by sex, the mean EEC volume was 609.89 ± 133.59 mm^3^ in males and 535.84 ± 90.3 mm^3^ in females, the mean EEC length was 17.07 ± 1.76 mm in males and 15 ± 1.77 mm in females, and the mean EEC width was 7.086 ± 1.715 mm in males and 7.401 ± 1.367 mm in females, while the mean TM area was 69.77 ± 7.4 mm^2^ in males and 66.17 ± 8.82 mm^2^ in females. Figure 3 shows the distribution of the EEC length, width, and volume and the TM area within the sample, highlighting the degree of variability and the range of measurements. Table 1 provides a comprehensive summary of the mean, standard deviation, and range for each parameter. Two-way ANOVA was conducted to assess the effects of ear side (left vs. right) and sex (male vs. female) on the EEC measurements, while variability was statistically quantified. The results revealed a significant main effect of ear side on all four parameters: length (F(24, 24) = 14.63, *p* < 0.0001), width (F(24, 24) = 10.78, *p* < 0.0001), volume (F(24, 24) = 15.04, *p* < 0.0001), and TM area (F(24, 24) = 6.104, *p* < 0.0001). In contrast, sex differences were only significant for length (F(1, 24) = 8.35, *p* = 0.0081). No significant sex effects were observed for width (F(1, 24) = 0.01379, *p* = 0.9075), volume (F(1, 24) = 1.144, *p* = 0.2954), or TM area (F(1, 24) = 0.8788, *p* = 0.3579) (Table 2).

### 3.2. Time Efficiency and Segmentation Accuracy of Manual and Automated Methods

We performed a comparison between manual and automated segmentation methods on 50 ears from 25 patients. Time efficiency was measured by recording the time required to complete each segmentation (i.e., each ear). The measurements of the length, width, and volume of the segmented 3D models were compared between the two methods to assess their geometric consistency. By comparing the results of manual segmentation with those of automated segmentation, we found a high degree of consistency.

**Figure 4:**
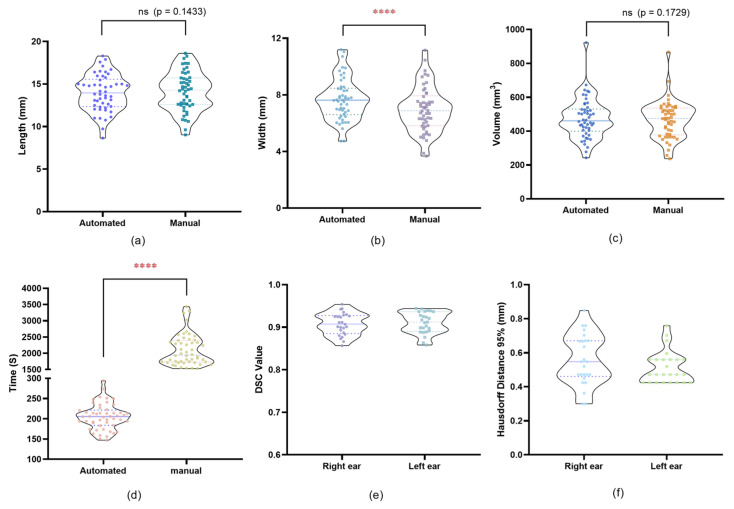
Comparison of manual segmentation method and automated segmentation plugin: (**a**) length comparison; (**b**) width comparison; (**c**) volume comparison; (**d**) time comparison; (**e**) DSC values; (**f**) 95% Hausdorff distance. Significance codes: **** *p* < 0.0001.

Both methods gave highly correlated results, with lengths measured by the manual method averaging 14.12 mm and those measured by the automated method averaging 13.97 mm. Similarly, the widths measured by the manual method averaged 6.98 mm, and the automated method averaged 7.67 mm. Paired *t*-tests showed no significant differences between length measurements (*p* = 0.1433). However, paired *t*-tests revealed a statistically significant difference between width measurement (*p* < 0.0001) (Figure 4a,b). Scatter plots comparing the volume measurements obtained by both methods showed a strong correlation. The volumes measured by the automated segmentation were consistently close to those measured manually, with no significant difference observed (*p* = 0.1729), indicating that the automated method reliably replicated the size obtained by manual segmentation (Figure 4c).

Time Efficiency: The average time spent on the manual segmentation process was significantly longer than the automated method. Specifically, the manual segmentation method took about 2103 s or 35.05 min per ear, whereas the automated segmentation plugin completed the same task in an average of 205 s or 3.42 min per ear. Statistical analysis using a paired *t*-test showed that the time difference between the two methods was statistically significant (*p* < 0.0001). This reduction in time demonstrates the efficiency of the automated plugin, making it particularly advantageous for clinical applications where time constraints are critical (Figure 4d).

**Figure 5:**
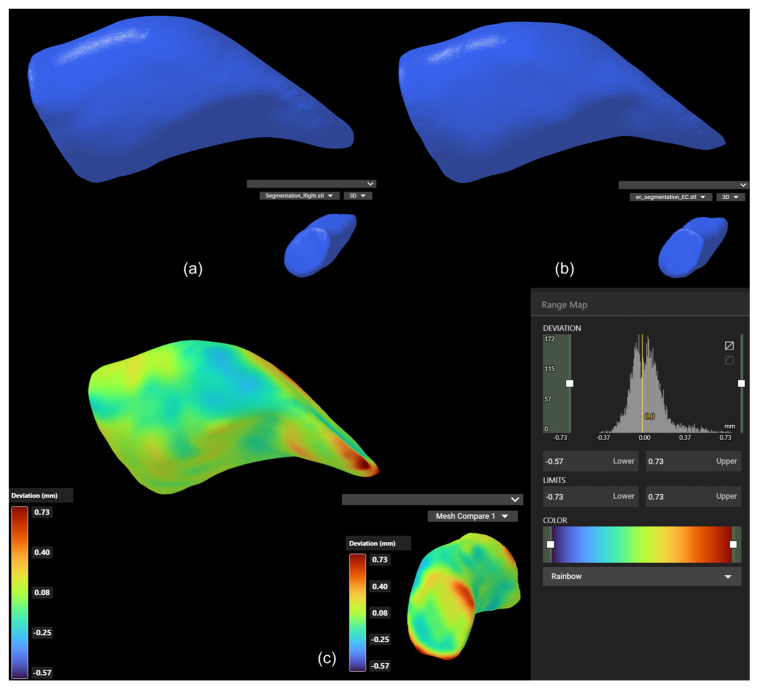
Comparison of 3D models segmented using manual and automated methods. (**a**) The manually segmented EEC 3D model. (**b**) The EEC 3D model segmented using the custom plugin. (**c**) Overlay of the two models, highlighting the high degree of overlap between the two segmentation methods.

Segmentation Accuracy and Consistency:

We evaluated the overlap and shape consistency between the 3D models generated by the manual and automated segmentation methods. The DSC was used as the main metric to quantify the overlap between the two 3D models of the EEC. In addition, the Hausdorff distance was calculated to evaluate the maximum deviation between corresponding points on the two model surfaces. The average DSC value for all samples was 0.90897658, and the average Hausdorff distance (95%) value was 0.53, indicating a high degree of overlap between the two methods (Figure 4e,f). We also conducted an overlapping comparison of the EEC 3D models generated by the two methods. The two 3D models were highly overlapping, which emphasized the segmentation consistency of the two methods (Figure 5).

EEC 3D models generated by both segmentation methods showed a close fit (Figure 5c), demonstrating the reliability and accuracy of our automated segmentation plugin.

### 3.3. Ex Vivo Comparative 3D-Printed EECI Implantations in Human Cadaver Temporal Bones

**Figure 6:**
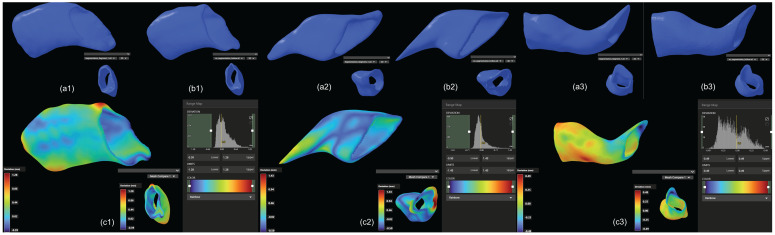
Comparison of 3D-printed EECI models generated from manual and automated segmentation methods. (**a1**–**a3**) Three-dimensional models of EECIs derived from manual segmentation for temporal bone specimens 1, 2, and 3, respectively. (**b1**–**b3**) Corresponding 3D models of EECIs generated using the custom automated segmentation plugin for the same specimens. (**c1**–**c3**) Overlapping models illustrating the high degree of concordance between EECIs generated by the two methods for each temporal bone specimen.

We first evaluated the consistency of EECIs generated from manual and automated segmentation. Overlapping comparisons of the EECI models demonstrated high concordance between the two segmentation methods, as illustrated in Figure 6.

**Figure 7:**
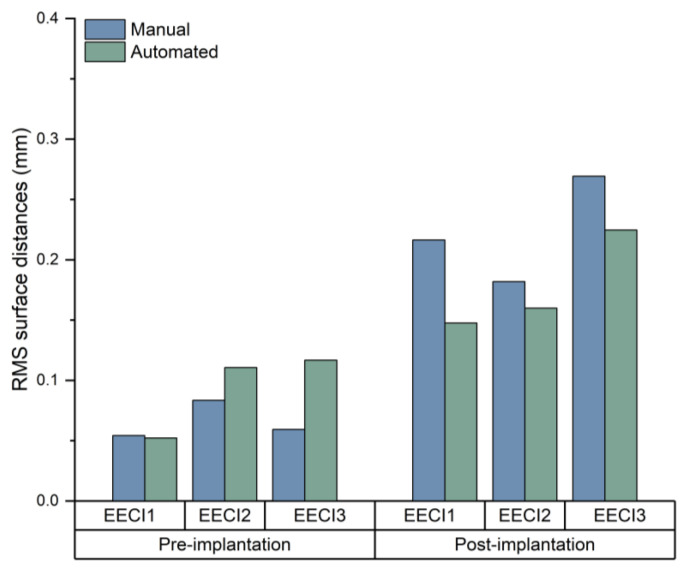
Comparative analysis of the RMS surface distances for EECI models derived by manual and automated segmentation relative to the EEC surface before and after implantation.

**Figure 8:**
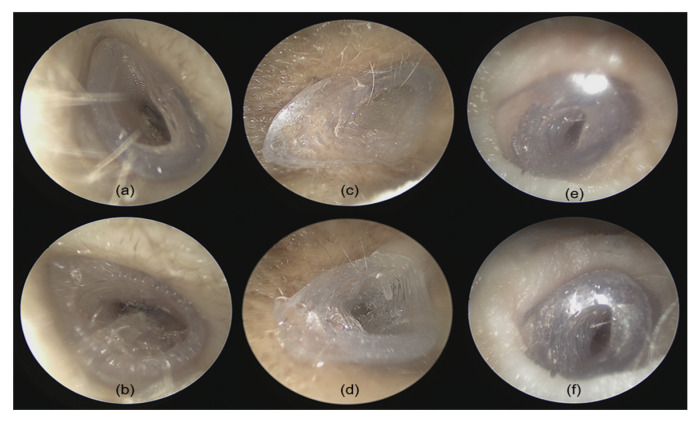
Post-insertion evaluation of EECIs derived from manual and automated segmentation methods in human cadaver temporal bones. (**a**,**c**,**e**) EECIs generated via manual segmentation, implanted in temporal bone specimens 1, 2, and 3, demonstrating proper fit, alignment, and accurate placement. (**b**,**d**,**f**) EECIs generated via automated plugin segmentation, implanted in temporal bone specimens 1, 2, and 3, showing comparable fit, placement, and alignment.

**Figure 9:**
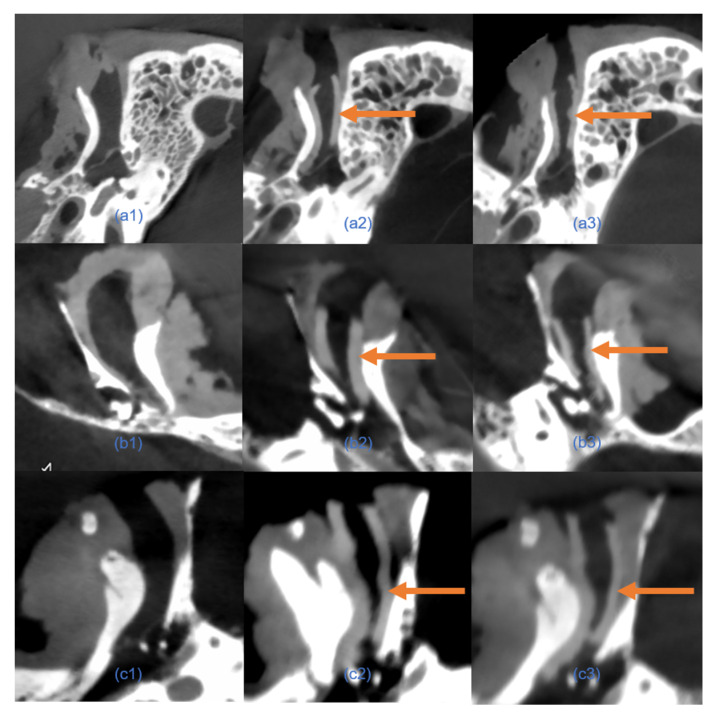
Comparative CBCT imaging of the human cadaver temporal bones before and after implantation of EECIs derived from manual and automated segmentation methods. (**a1**,**b1**,**c1**) Pre-implantation CT scans of temporal bone specimens 1, 2, and 3, respectively. (**a2**,**b2**,**c2**) Post-implantation CT scans showing the fit of EECIs generated by manual segmentation within temporal bone specimens 1, 2, and 3, respectively. (**a3**,**b3**,**c3**) Post-implantation CT scans showing the fit of EECIs generated by the automated segmentation plugin within temporal bone specimens 1, 2, and 3, respectively. Arrows indicate the position of the implanted EECIs.

**Figure 10:**
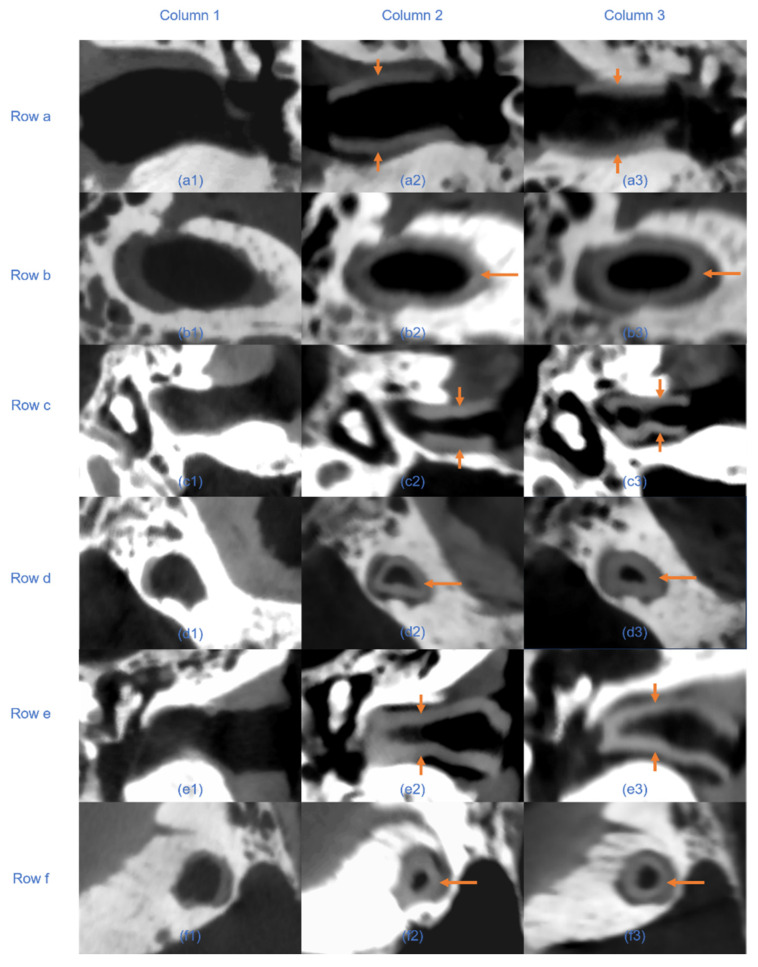
Axial and sagittal CBCT views of three human cadaver temporal bone specimens before and after implantation of EECIs derived from manual and automated segmentation. Columns: Column 1 shows pre-implantation scans; column 2 shows post-implantation with implants based on manual segmentation; column 3 shows post-implantation with plugin-based implants. Rows: **a**/**c**/**e** show axial views, and **b**/**d**/**f** show sagittal views of specimens 1, 2, and 3, respectively. Arrows indicate the position of the implanted EECIs. In axial views, bilateral arrows denote implant boundaries; in sagittal views, single arrows indicate overall implant alignment.

**Figure 11:**
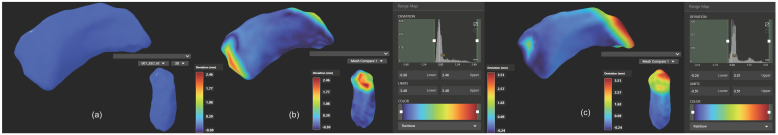
Overlapping comparison of EECI and EEC models derived from manual and automated segmentation before implantation. (**a**) The EEC model derived from human cadaver temporal bone specimen. (**b**) Overlapping EEC models with a manually segmented EECI model. (**c**) Overlapping EEC models with an automatically segmented EECI model. These visualizations highlight the spatial alignment and geometric consistency between EEC and EECI models generated through different segmentation methods, indicating the accuracy and conformity of the implants.

Quantitative analysis of the RMS surface distances revealed that the EECI models generated by manual segmentation exhibited RMS distances of 0.05 mm, 0.08 mm, and 0.06 mm relative to the EEC surface, while those generated by automated segmentation had RMS distances of 0.05 mm, 0.11 mm, and 0.12 mm. The results indicated a high degree of consistency and segmentation precision across both approaches (Figure 7). The otorhinolaryngologist rated all 3D-printed EECIs, regardless of segmentation method, as easily insertable and properly positioned. The performance evaluations for the insertion process are summarized in Table 3. Data are presented as mean values ± standard deviations. Specifically, all implants fit tightly against the bony walls of the EEC, with no visible gaps or misalignments along the edges (Figure 8). Post-insertion CBCT imaging further validated the fitting accuracy, demonstrating complete coverage of the EEC with no observable displacement or deformation of the implants (Figure 9 and Figure 10). In addition, the EECI models obtained by manual and automated segmentation before implantation demonstrated high spatial alignment and geometric consistency with the EEC model (Figure 11). Similarly, the comparison of EECI models before and after implantation, generated using both manual and automated segmentation methods, showed high geometric stability with minimal deformation, highlighting the accuracy and conformity of the implants. These findings indicate that the 3D printing and preparation process maintained high geometric accuracy, and no obvious deformation was observed after implantation. The RMS distances between the EEC model and the manually segmented EECI model after implantation were 0.22 mm, 0.18 mm, and 0.27 mm, while the RMS distances between the EEC model and the automatically segmented EECI model were 0.15 mm, 0.16 mm, and 0.22 mm, respectively (Figure 7). This further confirmed that the implant had high geometric stability and a close fit with the EEC.

## 4. Discussion

Anatomical variations in the EEC have been documented in previous studies. The length of the bony EEC has been reported to range widely, with a mean of 15.26 ± 2.37 mm (ranging from 1.45 to 24.10 mm) [13], whereas other findings suggest a shorter average length of approximately 9.61 mm [14]. The cross-sectional area of the cartilaginous portions of EEC has also been shown to vary depending on sex and body size, with larger individuals typically exhibiting greater dimensions [6]. Additionally, measurements of the TM have been reported, with mean values of 57.46 ± 16.23 mm^2^ for area, 9.54 ± 1.27 mm for vertical diameter, and 7.99 ± 1.08 mm for horizontal diameter in adult populations [15]. Recent studies highlight evolving perspectives on EEC anatomical variations. Whereas earlier work found no sex-based differences [16], recent CT analyses demonstrate that males exhibit significantly larger vertical and horizontal dimensions at the EAC entry and isthmus compared to females [10]. Conversely, both studies concur that no significant differences exist between left and right EEC dimensions or between individuals with and without chronic otitis media [10,16]. Such findings underscore the importance of standardized measurement protocols in future studies.

Considering these anatomical variations, ensuring accurate segmentation is crucial for the development of individualized EECIs. Unlike previous studies that focused exclusively on the bony EEC or on the cartilaginous portions of the EEC [13,14], our study extends the segmentation boundary to include both portions, allowing for a more comprehensive analysis of the EEC anatomy and its implications for implant design. The main aim of this study was to evaluate the efficiency and accuracy of the automated segmentation plugin compared to manual segmentation in the context of designing individualized implants for the EEC. Our results show that both manual and automated segmentation methods provide anatomically accurate EECIs with comparable fitting performance. The strong correlation observed between the two methods, particularly in volume and length measurements, further supports the reliability of automated segmentation. The reliability of the automated segmentation was further confirmed by the DSC values and HD 95%, showing minimal differences with the manual method. Our automated segmentation demonstrated superior performance compared to existing temporal bone CT segmentation methods. The mean DSC value of 0.909 in our study exceeds the reported DSC value of 0.821 for EEC segmentation in the recent literature [7]. Furthermore, the HD 95% of 0.53 mm achieved by our method represents an improvement over the 0.695 mm reported in prior work [7]. These enhancements likely stem from a key innovation: in our segmentation protocol, the implementation of adaptive thresholding algorithms tailored to the unique density gradients of EEC tissues.

Although the width measurements showed statistically significant differences between the manual and automated segmentation methods, this discrepancy arises from the inherent methodological differences. Manual segmentation calculates the width as the length of the line segment on the coronal plane passing through the midpoint of the long axis and perpendicular to it. In contrast, the custom plugin allows for precise delineation of the coronal plane that passes through the long axis’s midpoint and is orthogonal to the long axis, enabling accurate measurement of the longest line segment within this plane. This approach better aligns with the anatomical definition of width and highlights the superiority of automated segmentation in specific measurements. This study shows that both methods are capable of generating clinically viable implants for preventing EEC restenosis. And our automated segmentation plugin improves accuracy and repeatability of measurements. Meanwhile, the plugin provides a systematic method for the production strategy of individualized EECIs.

A key finding of this study was that the automated plugin significantly reduced segmentation time (up to 90% compared to the manual segmentation). The developed plugin achieves unprecedented efficiency gains through core innovations: Leveraging Python’s multiprocessing module, the plugin reduces segmentation time from 35.05 min (manual) to 3.42 min, representing a 90-time reduction—superior to the gains reported in previous research [7]. Reducing segmentation time is particularly critical in scenarios requiring rapid adjustments to implant designs during surgery or in the development of intraoperative 3D printing workflows. The integration of automated segmentation can thus streamline clinical workflows, reduce overall surgical time, and enhance patient outcomes by expediting implant customization and reducing perioperative risks. CT scans confirmed that the automated segmentation method accurately delineated EEC anatomy, providing a reliable basis for implant design. The resulting implant models demonstrated precise anatomical conformity and high geometric stability, both pre- and post-implantation (Figure 9). These findings validate the clinical utility of automated segmentation, particularly in streamlining implant customization for individual patients [17]. However, small differences in geometric measurements suggest that while automation can save considerable time, careful validation is still necessary, especially in complex anatomical areas.

This study also highlights the necessity for a personalized method to develop and design EECIs. Significant variability in EEC measurements between individuals, observed within the sample population, highlights the limitations of standardized implants in addressing patient-specific needs. Previous studies have explored the anatomical variability of the EEC using CT and MRI data, primarily focusing on general morphological characteristics [6,8,16]. However, existing CT-based studies have not specifically addressed EECI design or segmentation optimization. The segmentation approach in this study defines the external boundary using the zygomatic arch and mastoid as anatomical landmarks, rather than strictly confining it to the textbook-defined bony EEC. This boundary selection was motivated by clinical relevance, as EECIs are typically positioned in the bony region due to the lack of structural support in the cartilaginous portion [5,18]. Additionally, this approach ensures a standardized segmentation boundary for comparing manual and automated methods. Given that our segmentation spans both bony and cartilaginous components, this study provides the first comprehensive assessment of both structural regions, which may have implications for future implant designs. Furthermore, our study is the first to investigate EEC individuality in the context of implant development, employing an automated segmentation approach to enhance precision and reproducibility. This distinction highlights the novelty and clinical relevance of our findings.

Current surgical treatments for preventing EEC restenosis typically involve the use of stents, molds, or tubes to maintain the canal’s patency postoperatively [4,19,20]. However, these methods often present challenges such as improper fit, displacement, and patient discomfort, which can compromise long-term outcomes. In contrast, individualized EECIs offer a tailored solution, providing a precise fit to the patient’s unique anatomy. This reduces the risk of restenosis by ensuring optimal contact with the bony canal walls, minimizing gaps or misalignment, and promoting stable healing. The findings presented in this study have the potential to significantly reduce the incidence of EEC restenosis by providing a more targeted treatment method, ultimately improving patient outcomes. A previous study has suggested that customized drug-eluting implants, such as EECI, may influence the retention of therapeutic agents within the EEC [5]. However, direct in vivo evidence is still lacking, highlighting the need for further research to confirm these findings in larger clinical studies. This prolonged drug retention may further enhance the therapeutic efficacy of drug-eluting implants, thus providing an additional advantage in clinical applications.

To date, automation in EEC segmentation and implant design remains underexplored, largely due to the lack of training datasets tailored for otological applications [7]. Although artificial intelligence (AI)-based methods are increasingly used for medical image segmentation, their application in 3D anatomical data is still limited by the need for extensive computational resources and high-quality training datasets [21,22,23]. In this context, our custom plugin provides a powerful alternative to manual segmentation of the EECs. Furthermore, the tool offers the potential to generate high-quality training data for future standalone, fully automated AI solutions, thereby facilitating wider adoption of automation in otology. This dual capability highlights its value as both a practical solution for current workflows and as a foundation for driving innovation in automated segmentation research.

The clinical significance of this study is profound. By integrating an automated segmentation plugin into the clinical workflow, clinicians can speed up the implant design process, potentially improving patient outcomes by reducing procedure time and ensuring a better-fitting implant. However, this study also highlights the importance of combining automated methods with expert verification to ensure the accuracy of the final design. The findings presented in this study have the potential to significantly reduce the incidence of EEC restenosis by providing a more targeted treatment method, ultimately improving patient outcomes. Upcoming studies from our research group are evaluating the use of EECI in preventing EEC restenosis. Furthermore, the methods developed here could be adapted and applied to other areas of otology, expanding the impact of our work. This line of research aims to provide further evidence for the clinical benefits of these individualized implants and explore their potential to improve long-term patient outcomes.

Despite promising results, this study has several limitations. First, the sample size was relatively small, which may limit the generalizability of the findings. Although 50 ears from 25 patients were included, future research should focus on expanding the dataset to include more diverse anatomical variations and larger population cohorts. Additionally, while the segmentation plugin demonstrated accuracy and efficiency in retrospective CBCT datasets and ex vivo fitting tests, it was not validated in real clinical procedures. These experimental settings do not fully capture soft tissue behavior, biological variability, or the practical challenges of in vivo implantation. Therefore, future work should include prospective clinical studies using real patient data, with intraoperative segmentation-assisted implantation and postoperative follow-up to evaluate implant stability, patient comfort, and efficacy in restenosis prevention.

Furthermore, the limited soft tissue contrast inherent to CBCE imaging may affect the precise identification of structures, such as the TM boundary, although our segmentation strategy mitigates this limitation through spatial constraints and intensity-based refinement. The current segmentation framework could also benefit from the integration of advanced imaging modalities and the incorporation of machine learning techniques to enable real-time and fully automated customization workflows [7]. By addressing these challenges, the proposed method can be further refined and translated into clinical practice, supporting broader applications in individualized otologic surgery and other anatomically complex interventions.

## 5. Conclusions

This study demonstrates that automated segmentation methods provide a viable alternative to manual segmentation for the design of individualized implants for the EEC. Geometric and volumetric analyses confirmed that the automated method significantly reduces segmentation time while maintaining high accuracy. The observed differences in EEC measurements among the patient population emphasize the need for individualized implant designs, rather than relying on standardized models.

Integrating automated methods into clinical practice could streamline the clinical workflow, reduce the time required for implant design, and potentially improve patient outcomes. However, to ensure the highest accuracy, it remains crucial to combine automated segmentation with expert validation. Future studies should continue to refine these methods and expand their application to different patient demographics and anatomical regions.

These findings provide a strong foundation for the continued development of automated segmentation tools in the field of personalized medicine, especially in the design of customized implants. As technology advances, the potential for fully automated, high-precision implant design grows, promising significant improvements in surgical outcomes and patient care.

## Figures and Tables

**Table 1:** Summary of the EEC measurements.

Measurement	Mean	SD	Min	Max	Range
Length (mm)	15.91	2.063	10.75	19.99	9.24
Width (mm)	7.262	1.554	3.634	11.06	7.426
Volume (mm^3^)	568.4	118.5	308.8	999.7	690.9
TM Area (mm^2^)	67.75	8.501	49.56	88.28	38.72

**Table 2:** Two-way ANOVA results for the effects of ear side and sex on the EEC measurements.

Measurement	Ear Side			Sex		
	F-Statistic	*p*-Value	Significance	F-Statistic	*p*-Value	Significance
Length (mm)	14.63	<0.0001	****	8.35	0.0081	**
Width (mm)	10.78	<0.0001	****	0.01379	0.9075	ns
Volume (mm^3^)	15.04	<0.0001	****	1.144	0.2954	ns
TM Area (mm^2^)	6.104	<0.0001	****	0.8788	0.3579	ns

Note: ** *p* < 0.01; **** *p* < 0.0001, based on two-way ANOVA results.

**Table 3:** Performance evaluation of the implantation process for EECIs derived from manual and automated segmentation methods.

Specimen	Segmentation Method	Handling Difficulty (1–6)	Implantation Time (s)	Endoscopic Fitting Accuracy (1–6)	CBCT Fitting Accuracy (1–6)
1	Manual	1	21.5 ± 8.5	2	1
1	Automated	1	10.56 ± 3.56	1	1
2	Manual	1	33 ± 22	1	2
2	Automated	1	8.68 ± 1.68	1	1
3	Manual	2	38.5 ± 11.5	2	1
3	Automated	1	19.5 ± 1.5	2	1

## Data Availability

The original contributions presented in this study are included in the article. Further inquiries can be directed to the corresponding author.

## Abbreviations

The following abbreviations are used in this manuscript:

EEC	External ear canal;
EECI	External ear canal implant;
HRCT	High-resolution computed tomography;
3D	Three dimensional;
TM	Tympanic membrane;
CBCT	Cone-beam computed tomography;
CT	Computed tomography;
DICOM	Digital Imaging and Communications in Medicine;
DSC	Dice similarity coefficient;
HD 95%	The 95th percentile Hausdorff distance;
STL	Standard tessellation language;
RMS	Root mean square;
ANOVA	Analysis of variance.
